# Enhancing Case Formulation Competence in Novice Counselors: A ChatGPT-Assisted Approach Using the 4P Model

**DOI:** 10.3390/bs16081315

**Published:** 2026-08-03

**Authors:** Yanshan Dai, Han Han, Yongze Xu

**Affiliations:** 1 Faculty of Psychology, Beijing Normal University, Beijing 100875, China; 202228061377@mail.bnu.edu.cn (Y.D.); 202521061125@mail.bnu.edu.cn (H.H.); 2 Bay Area School of Applied Psychological Sciences, Beijing Normal University at Zhuhai, No. 18 Tangjiawan Jinfeng Road, Xiangzhou District, Zhuhai 519087, China; 3 Beijing Key Laboratory of Applied Experimental Psychology, National Demonstration Center for Experimental Psychology Education (Beijing Normal University), Faculty of Psychology, Beijing Normal University, Beijing 100875, China

**Keywords:** ChatGPT, case formulation, novice counselors, 4P model, counselor training

## Abstract

With the rapid advancement of artificial intelligence (AI) technology, various scientific research fields are exploring the application of potential of new technologies. Counseling psychology, closely intertwined with natural language processing, is one of the most suitable domains for such exploration. This study focuses on investigating the effectiveness of large language models (LLMs) in assisting novice counselors with case-formulation processes. Based on supervisors’ ratings of novice counselors’ performance before and after using LLMs, the results indicate that AI assistance significantly enhances the comprehensiveness and coherence of case formulation, while its effects on complexity and specificity are not statistically significant. Additionally, through novice counselors’ subjective evaluations of the AI-assisted process, the value of AI tools in broadening perspectives and facilitating convenient operations is identified. Overall, the findings suggest that AI can moderately assist novice counselors in understanding clients. However, to better integrate AI with professional practice and improve case formulation competence, it is essential for novice counselors to maintain critical thinking and strengthen their learning of counseling theories and techniques during AI application.

## 1. Introduction

Recent advances in Artificial-Intelligence-Generated Content (AIGC) and Natural Language Processing (NLP) technologies have created new opportunities for the field of mental health. Within the domain of psychological counseling, recent research has confirmed that AI systems built on GPT-based technologies can effectively analyze counseling transcripts and enhance the professional competencies of psychological counselors (Liu et al., 2023). Empirical evidence has demonstrated that AI exhibits considerable potential in the identification of mental health disorders (Khurana et al., 2023; Uban et al., 2021; Zhang et al., 2022), the formulation of personalized treatment regimens (Mohsin et al., 2023; Sarker, 2021; Thieme et al., 2023), and the development of virtual therapists (Darnell et al., 2022; Durden et al., 2023). As the applications of AIGC in mental health continue to expand, its applicable scenarios are still being further explored. Case conceptualization, a key component of psychological counseling, pertains to the systematic comprehension of clients’ problems and the formulation of intervention strategies. Exploring the role of AIGC in case conceptualization is therefore of great significance for improving the counseling proficiency of novice counselors.

Case conceptualization occupies a central position in the field of psychological counseling. As a core component that ensures the effective progress of the counseling process and facilitates clients’ problem-solving, it is regarded as one of the essential professional competencies for psychological counselors (An, 2006), and it plays a critical role in accurately understanding clients’ conditions and formulating targeted intervention strategies. By definition, case conceptualization encompasses in-depth understanding and reasoned inferences regarding the relationships between clients’ problems and symptom synthesis that enables the prediction of clients’ behavioral and psychological responses (Turkat, 1985). In actual counseling practice, counselors are required to continuously verify, adjust and refine these understandings and hypotheses (An et al., 2015). However, notable disparities exist between novice and senior counselors in the practical application of case conceptualization (Martin et al., 1989). In terms of client information collection, senior counselors, leveraging their extensive clinical experience, can typically obtain all the information required for complete case conceptualization during the initial interview. In contrast, novice counselors often need to conduct four or more sessions to effectively accomplish this task (Baer, 2004). Regarding the procedural execution of case conceptualization, novice counselors tend to focus on superficial details, thus generating a multitude of idiosyncratic yet fragmented organizing constructs (Kivlighan & Quigley, 1991; Martin et al., 1989; Mayfield et al., 1999). In comparison, experienced counselors are adept at employing fewer but more generalizable organizing constructs that not only encompass case-specific details but also establish broader and more in-depth conceptual connections (Mayfield et al., 1999). These differences directly correspond to the four dimensions of the Case Formulation Quality (CFQ) scale: novices’ tendency toward fragmented, superficial details reflects deficits in comprehensiveness and coherence, while their difficulty in establishing broad conceptual connections indicates limitations in complexity and specificity.

The definition of novice counselors (or novice therapists) comes from systematic research into the professional development trajectory of counseling practitioners. The six-phase model of counselor development proposed by Rønnestad and Skovholt (2003) posits that differences between counselors at distinct developmental stages are reflected in their helping strategies and clinical performance. Novice counselors correspond to the “beginning professional” phase within this model, a stage during which individuals have just commenced systematic professional training and are committed to mastering counseling techniques and forging their professional identity (Xu & Hou, 2010). Within the normative framework established by the Chinese Psychological Society (CPS), psychological counselors are further categorized into three levels: assistant psychological counselors, certified psychological counselors, and clinical supervisors. Practitioners who have not yet met the qualification criteria for certified psychological counselors are defined as novice counselors or certified assistant psychological counselors. In existing empirical research, the term novice counselors generally refer to psychology students with a foundational grasp of counseling theories and limited clinical experience; this designation is particularly applicable to junior postgraduate students majoring in psychological counseling who have completed theoretical coursework in counseling (An et al., 2015), as well as undergraduate students in psychology programs (Al-Darmaki, 2004).

Psychological counseling encompasses a diverse array of theoretical orientations, including cognitive behavioral therapy, psychoanalysis, humanistic therapy, and integrative approaches. Each orientation is underpinned by a unique theoretical foundation, has developed its own proprietary therapeutic methods and techniques, and has further derived a corresponding approach to case conceptualization (Bucci et al., 2016). Among these frameworks, the 4P model has garnered extensive attention in clinical practice due to its notable advantages. This model integrates biological, psychological, and social factors and centers on four core components: predisposing factors, which explore why an individual is vulnerable to the problem; precipitating factors, which analyze why the problem has emerged at this specific time; perpetuating factors, which examine why the problem persists; and protective factors, which consider why the condition has not deteriorated (Bolton, 2014; Winters et al., 2007; Wright et al., 2019). By comprehensively considering these four dimensions, the 4P model enables clinicians to conduct a holistic case analysis and thereby formulate evidence-based therapeutic interventions. With its capacity for multi-factor integration, the 4P model is a comprehensive, multidimensional analytical framework and has achieved widespread recognition within the field (Guerrero et al., 2003; McClain et al., 2004). In the present study, the integrative 4P model was adopted as the theoretical underpinning for AI-driven case conceptualization, and AI prompt engineering was designed on the basis of this model to generate case conceptualization content. To assess novice counselors’ acceptance of the process and content of AI-generated case conceptualization, the Technology Acceptance Model (TAM) was employed in the research design.

The Technology Acceptance Model (TAM) is designed to assess users’ acceptance of new technologies (Davis, 1989), with its core constructs being Perceived Ease of Use (PEU) and Perceived Usefulness (PU). This model aims to elucidate the factors underlying users’ rejection of information technologies and how system characteristics shape users’ behavioral intentions to adopt such technologies. Furthermore, TAM is one of the most mature and robust theoretical models in the field of information systems applications, owing to its ability to accurately predict and explain users’ willingness to accept and actual usage behaviors of a given system. It is widely applied to investigate how external variables indirectly influence system usage by impacting perceived ease of use and perceived usefulness. Empirical evidence has demonstrated that TAM can effectively evaluate users’ experience with and acceptance of innovative technologies such as ChatGPT (Barakat et al., 2024; Sweeney & Soutar, 2001; Zou & Huang, 2023). These two frameworks serve complementary roles in our design: the 4P model functions as the analytical framework for structuring case conceptualization content generation, while TAM serves as the evaluative framework for assessing novice counselors’ acceptance and subjective experience of AI assistance.

In summary, to examine the ability of artificial intelligence (AI) to perform case conceptualization independently and explore its potential to enhance the quality of novice counselors’ case conceptualization outcomes, two core hypotheses are proposed in this study:

**H1.**  *AI is capable of completing case conceptualization independently.*

**H2.**  *AI can assist novice counselors in improving the quality of their case conceptualization outcomes.*

Specifically, the study was divided into three phases: a pilot study, Experiment 1, and Experiment 2. The primary objective of the pilot study was to identify effective prompts for input into large language models (LLMs). The specific content of the prompts was developed by integrating output format requirements based on the 4P model with a well-established prompt framework, and the effectiveness of the prompts was comprehensively evaluated according to the validity and reproducibility of the model’s output. Experiment 1 was designed to examine AI’s ability to perform case conceptualization independently. AI was instructed to conduct case conceptualization analyses on classic publicly available cases, and the quality of the resulting analyses was rated by clinical supervisors. Hypothesis 1 was tested by determining whether these ratings met the predefined quality criteria. Experiment 2 aimed to explore AI’s potential to improve novice counselors’ case conceptualization outcomes. By comparing the differences in the quality ratings of novice counselors’ case conceptualization work before and after AI-assisted practice across two scenarios (classic cases and real-world cases), Hypothesis 2 was empirically tested.

## 2. Methods

This study employed a three-phase design to test the two core hypotheses, respectively. Experiment 1 was designed to test H1 (AI’s independent ability to complete case conceptualization), establishing whether AI-generated formulations could reach a passing quality standard. Experiment 2 was designed to test H2 (AI’s effectiveness in improving novice counselors’ case conceptualization performance), comparing novices’ work before and after AI-assisted support. The pilot study preceded both experiments to establish the necessary prompt engineering infrastructure.

### 2.1. Participants

#### 2.1.1. Large Language Model

During the conduct of this study, LLM technology was still in a phase of active development; the maturity of multimodal information processing functionality remained to be enhanced, and output quality was highly dependent on prompt engineering. Careful prompt design was therefore critical to improving the quality of AI-generated responses. In the pilot study phase, therefore, we conducted tests on mainstream LLMs including Qianfan, txyz.ai, and ChatPDF, with a focus on two core performance metrics: document reading capability and task completion efficiency for long text. The results revealed that these models generally exhibited technical bottlenecks in practical applications, such as insufficient accuracy in document parsing and a lack of contextual coherence in processing long text. Following multiple rounds of comparative testing and comprehensive analysis, the GPT-4o model developed by OpenAI (specifically, the gpt-4o-2024-05-13 checkpoint) was ultimately selected as the unified platform for experimental implementation, as it demonstrated clear advantages in multimodal information processing and the execution of long-sequence tasks. The GPT-4o model was consequently adopted to complete all experimental tasks across the study. A consistent model version was used for all three experiments, with the experimental period spanning from 16 to 21 July 2024. Given the rapid iteration of LLMs, the performance observed in this study reflects the model version available at the time of the experiment; subsequent versions may yield different results.

#### 2.1.2. Participants

A total of five novice counselors were recruited as participants to take part in the experiments. The inclusion criteria for novice counselors were as follows: (a) being current postgraduate students majoring in psychological counseling or graduates within one year of graduation; (b) having limited clinical counseling experience (≤200 h of clinical practice), consistent with the developmental stage of trainees in the early professional phase (Rønnestad & Skovholt, 2003). Demographic and professional characteristics of the participants are presented in Table 1. Given the exploratory nature of this study and the small sample size (*n* = 5), findings should be interpreted with appropriate caution.

### 2.2. Experimental Materials

The experimental materials comprised two components: a publicly available dataset and a self-developed dataset. The publicly available dataset consisted of counseling transcripts of cases conducted by senior psychological counselors, while the self-developed dataset included counseling transcripts of cases completed by the participants themselves. Given that senior counselors outperform novice counselors in the application of counseling techniques and information integration abilities (Baer, 2004), and are thus able to collect sufficient and critical client information in fewer sessions, the publicly available dataset for both Experiment 1 and Experiment 2 uniformly included transcripts from the first three counseling sessions with the same client, whereas the self-developed dataset uniformly included transcripts from the first six counseling sessions.

Specifically, the publicly available dataset was selected from the Counseling and Psychotherapy Transcripts Series published by Alexander Street (AS) Press. Its authority is evidenced by the following aspects: the database contains 2000 original counseling records directly provided by professional institutions, with only sensitive information undergoing standard anonymization; text processing strictly adheres to the ethical guidelines of the American Psychological Association (Psychology & Nursing Collection, 2024); all materials have been reviewed by the AS Editorial Board, which comprises practicing clinicians and researchers (Counseling & Therapy in Video Library, 2019). In consideration of the competency level of novice counselors, two individual counseling cases without manifestations of mental disorders were selected from the database as the experimental materials. The self-developed dataset was derived from counseling transcripts of the participants’ most recently completed cases prior to their participation in the study, and informed consent was obtained from clients prior to data usage.

The verbatim transcripts varied in length across cases: Case A (Client110) comprised 20,440 words, Case B (Client018) comprised 31,637 words (both across the first three sessions), and Case C comprised 40,983 words across all six sessions. Further details are presented in Table 2.

### 2.3. Experimental Instruments

The instruments employed in this study included three components: a prompt framework, research questionnaires, and expert raters. The research questionnaires were further divided into self-reported and other-reported versions, with a total of three questionnaires and one rating manual developed for the study.

#### 2.3.1. Prompt Framework

In the pilot study, three prompt frameworks were utilized: LangGPT (M. Wang et al., 2024), BROKE (Chen & Li, 2023), and Co-star (GovTech Data Science & AI Division, 2023). Within all these frameworks, the theoretical knowledge of the 4P model and task rules for a three-stage output format were embedded. The prompts designed based on these three frameworks were jointly adopted for both Experiment 1 and Experiment 2.

#### 2.3.2. Case Formulation Quality Questionnaire and Rating Manual

The Case Formulation Quality (CFQ) scale and its accompanying Case Formulation Quality Rating Manual (CFQRM) were adopted as the measurement instruments for assessing the quality of case conceptualization in this study. The CFQ comprises four dimensions: comprehensiveness (the presence and quality of coverage of problems, etiology, and explanatory mechanisms), coherence (the logical and causal connections among formulation components), complexity (the degree of differentiation and integration of multiple facets of functioning), and specificity (the degree of individualization, precision of language, and clarity of treatment focus). Its detailed scoring criteria are incorporated in the CFQRM, which renders the CFQ one of the few validated assessment methods for case conceptualization with empirical validity data (Baer, 2004).

The CFQ uses a 5-point Likert scale, with scores ranging from 1 to 5 corresponding to Absent, Inadequate, Adequate, Good, and Excellent, respectively. The full CFQ scale is presented in Appendix A. Based on the detailed definitions of each rating in the CFQRM, a score of 2 (Inadequate) is defined as poor descriptive quality, and a score of 3 (Adequate) is defined as appropriate description. Thus, a score of 3 was established as the passing criterion for the quality of case conceptualization. The detailed scoring criteria of the CFQRM are described in Baer (2004; see Appendix B).

#### 2.3.3. Perceived Usefulness and Perceived Ease of Use Scales

To measure novice counselors’ subjective experiences of GPT-4o outputs, this study adopted the core constructs of Perceived Usefulness (PU) and Perceived Ease of Use (PEU) from the Technology Acceptance Model (TAM) proposed by Davis (1989). In the present study, PU was defined as novice counselors’ subjective evaluation of the validity and practical utility of AI-generated case conceptualization content following AI usage; PEU was defined as their perception of the intuitiveness and user-friendliness of the AI’s operational interface during usage, as well as their subjective assessment of the ease or difficulty of AI utilization.

We adapted the original PU questionnaire in a targeted manner based on the characteristics of case conceptualization tasks and the operational features of GPT-4o. Specifically, items related to completing tasks faster and improving efficiency in the original questionnaire were merged to emphasize the dimension of work efficiency. Meanwhile, in accordance with the task characteristics of this study, items pertaining to job performance in the original questionnaire were revised to assess whether ChatGPT assisted participants in engaging in more in-depth thinking during the case conceptualization process. Furthermore, items related to productivity were eliminated given that case conceptualization is not directly associated with this construct. For the PEU scale, the item measuring ease of mastery was excluded because novice counselors only used ChatGPT on a single occasion.

Sample items from the adapted scales include: “ChatGPT can shorten the time I need to complete case conceptualization and improve efficiency” (PU) and “I find it easy to operate ChatGPT to analyze transcripts and generate case conceptualizations” (PEU). Both scales are presented in Appendix B. Both the PU and PEU scales adopted a 5-point Likert response format, with higher scores indicating greater agreement with the corresponding statements. Scores ranging from 1 to 5 were labeled as Strongly Disagree, Disagree, Neutral, Agree, and Strongly Agree, respectively.

#### 2.3.4. Raters

Five certified psychologists accredited by the Registration System of the Chinese Psychological Society (CPS) were recruited to conduct ratings for the study. All raters had accumulated more than 1500 h of clinical counseling experience and had received, and continued to participate in, systematic professional supervisory training. The supervisors had an average of 7 years of clinical practice experience, with their theoretical orientations including psychodynamic therapy *(n* = 2), narrative therapy (*n* = 2), and integrative therapy (*n* = 1).

Case conceptualization for each case was rated on the CFQ by two independent supervisors. The specific allocation of supervisors to cases is presented in Table 3.

### 2.4. Study Procedure

**Figure 1:**
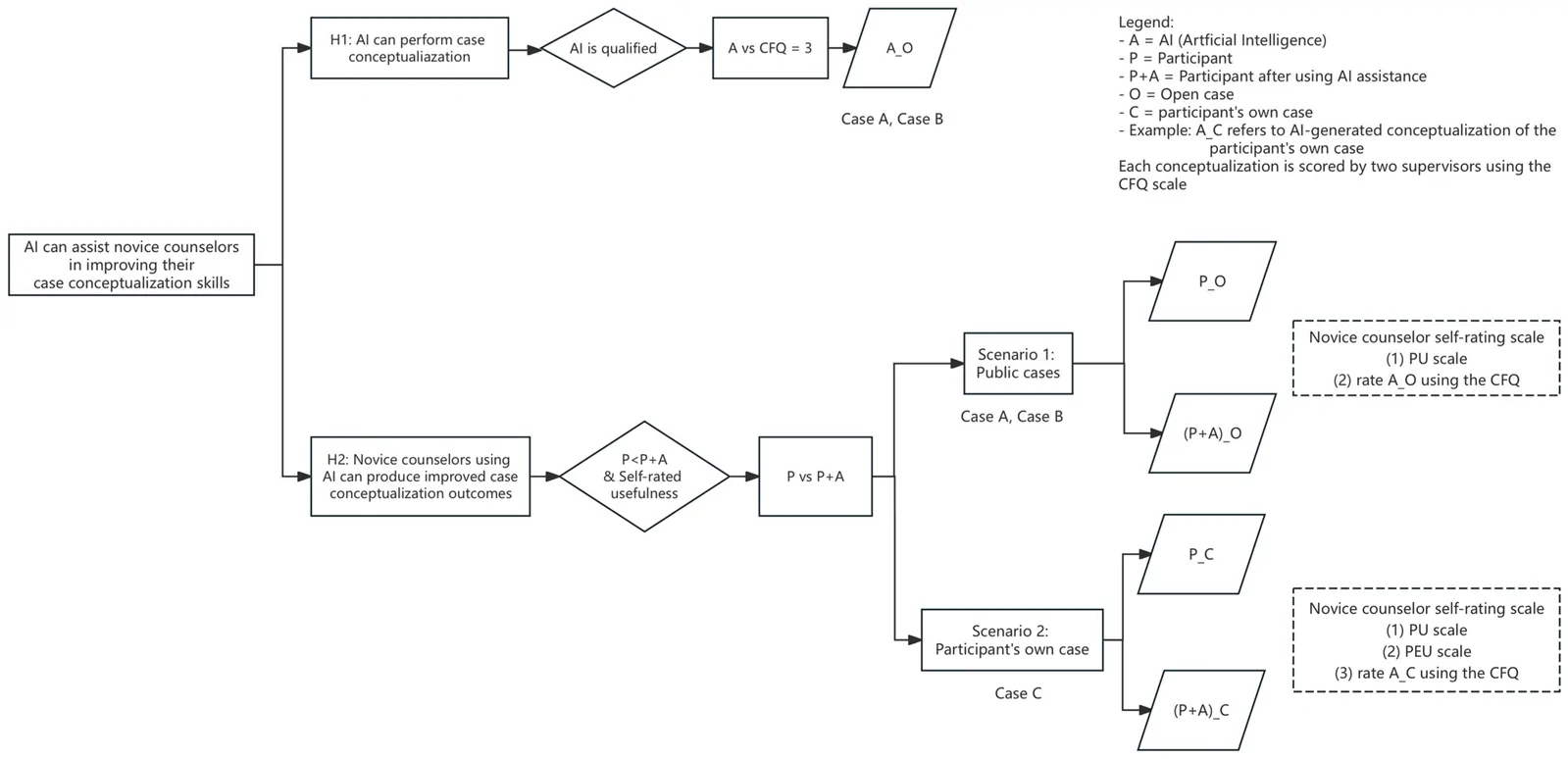
Study flowchart.

The research workflow of the present study is illustrated in Figure 1. Table 4 summarizes the participants, cases, AI involvement, and outcome measures across the three phases. In the assessment phase, clinical supervisors conducted a double-blind rating of the AI-generated and novice counselor-developed case conceptualization content for the corresponding cases, with the case conceptualization materials for each case randomly ordered prior to rating.

#### 2.4.1. Pilot Study

We systematically designed three sets of prompts based on three prompt frameworks: LangGPT, BROKE, and Co-star. Given that the frameworks exhibit the characteristics of high conciseness, systematicity, and replicability, the developed prompts were directly reusable and demonstrated superior performance compared to unstructured self-constructed prompts. The finalized prompts, developed from all three frameworks were applied in both Experiment 1 and Experiment 2, with the complete prompt texts presented in Appendix C.

The prompt design process was guided by the prompt pattern theory proposed by White et al. (2023), and the systematic design was primarily carried out across four dimensions: role positioning, reference integration, interaction mechanism construction, and output specification formulation. The prompts were evaluated and iteratively optimized by assessing three dimensions of AI outputs: completeness, instruction compliance, and replicability. The detailed design logic is presented in Table 5.

#### 2.4.2. Experiment 1: AI’s Ability to Perform Case Conceptualization

To control for variability in AI interaction quality arising from users’ query phrasing and academic backgrounds (Marvin et al., 2024; Chiu, 2024; Wu et al., 2023), the researchers managed all AI operations in Experiment 1 and Scenario 1 of Experiment 2. Specifically, for each of the three prompt frameworks, the researchers generated three outputs per case, selected the highest-quality one for rating, and collated all outputs for participants’ reference. In contrast, participants in Scenario 2 operated ChatGPT independently to complete AI-assisted process.

Based on the prompts derived from the three prompt frameworks developed in the pilot study, GPT-4o was used to conduct case conceptualization analyses on the counseling transcripts of Case A and Case B. Three outputs were generated for each prompt framework per case, and the highest-quality output among the three was selected for CFQ rating by two independent clinical supervisors, who also provided subjective comments. AI’s capacity to independently perform case conceptualization was determined by whether the resulting CFQ ratings met the predefined passing criterion.

#### 2.4.3. Experiment 2: The Effect of AI-Assisted Support on Novice Counselors’ Case Conceptualization Competence

The supportive effects of AI on novice counselors were investigated across two distinct scenarios to explore its performance in different contextual settings: Scenario 1 examined novice counselors’ performance in classic case practice, and Scenario 2 explored their use of AI in real-world clinical practice. In both scenarios, all participants first independently developed an initial draft of case conceptualization, and then revised and refined a final draft with the assistance of AI tools.

The two scenarios differed in AI operation procedures (as specified at the beginning of Section 2.4.2): in Scenario 1, AI outputs were researcher-managed, with collated outputs from the three prompt frameworks provided to participants for reference. This design was implemented because the quality of AI-generated content is influenced by users’ query phrasing (Marvin et al., 2024) and academic backgrounds (Chiu, 2024; Wu et al., 2023). In Scenario 2, participants operated ChatGPT independently to complete the AI-assisted process. In practice, all three prompt frameworks were consulted by the participant during the AI-assisted revision process.

Both hetero-evaluation and self-evaluation were adopted as assessment approaches. For the hetero-evaluation component: the quality of case conceptualization was primarily assessed by clinical supervisors using the CFQ scale, with accompanying subjective comments. The effect of AI-assisted support on improving novice counselors’ case conceptualization competence was determined by comparing the CFQ scores of their case conceptualization work before and after AI intervention. For the self-evaluation component: all participants completed the CFQ and PU scales based on the AI-generated case conceptualization content as a supplement to their subjective perceptions. Since only participants in Scenario 2 engaged in the hands-on operation of AI, only this group of participants was required to complete the PEU scale.

## 3. Results

### 3.1. Experiment 1

#### 3.1.1. Reliability and Validity Analysis

The reliability and validity of the Case Formulation Quality (CFQ) scale were first examined in the present study. Based on the rating data from 32 clinical supervisor evaluations, the results indicated good internal consistency (α = 0.90). The data were suitable for exploratory factor analysis, as supported by a Kaiser–Meyer–Olkin (KMO) value of 0.76 and a significant Bartlett’s Test of Sphericity (*p* < 0.001). Additionally, the unifactorial structure of the CFQ accounted for 76.36% of the total variance.

#### 3.1.2. Inter-Rater Consistency

The root mean square deviation (RMSD) was employed to assess inter-rater consistency across the four dimensions of the CFQ. Results showed highest consistency for the coherence (RMSD = 0.829), followed by comprehensiveness (RMSD = 1.090). Consistency was relatively lower for the complexity and specificity dimensions (RMSD = 1.118/1.436). Notably, the discrepancies across all dimensions fell within an acceptable range.

#### 3.1.3. Clinical Supervisors’ Ratings of AI-Generated Case Conceptualization Quality

To evaluate the quality of AI-generated case conceptualization, a 2 (case) × 3 (prompt framework) repeated-measures analysis of variance (ANOVA) was conducted. As shown in Table 6, the results indicated no significant main effects or interaction effects across the four dimensions of the CFQ (*p* > 0.05), demonstrating that the quality of AI-generated case conceptualization was not affected by prompt framework or case type.

Further one-sample *t*-tests (test value = 3) were conducted to determine whether AI-generated case conceptualizations reached the predefined passing threshold. As shown in Table 7, on the coherence dimension, AI performance reached a statistically significant passing level (M = 3.42, SD = 0.76, t = 1.82, *p* = 0.01). On the specificity dimension, the mean score exactly met the passing threshold (M = 3.00, SD = 0.82, t = 0.00, *p* = 1.000). Although the comprehensiveness dimension (M = 3.25) and complexity dimension (M = 2.92) did not reach statistical significance, their actual mean values were close to the passing threshold (mean difference < 0.1). Collectively, these findings suggest that the overall quality of AI-generated case conceptualization met an acceptable level, with the highest performance on coherence and relatively weaker performance solely on complexity. Thus, H1 is partially supported: AI is capable of independently completing case conceptualization at a passing level on the coherence and specificity dimensions, though performance on comprehensiveness and complexity did not reach the passing threshold.

#### 3.1.4. Qualitative Analysis of Supervisors’ Feedback on AI-Generated Outputs

To complement the quantitative findings, one author reviewed all supervisors’ open-ended comments and conducted an inductive categorization of the extracted observations. Specifically, discrete observations were first identified from each supervisor’s comments; these observations were then grouped into subcategories based on content similarity; finally, the subcategories were further consolidated into broader thematic categories. Only themes mentioned by at least two supervisors were retained for reporting, to ensure that the reported themes reflected relatively consistent patterns across evaluators. Through this inductive categorization, supervisors’ evaluations of AI-generated case conceptualizations yielded three major dimensions: structural strengths (information organization and targeted focus), depth-related limitations (superficial analysis and weak emotional connection), and theoretical integration challenges (theoretical overcrowding and mismatch with intervention plans).

A qualitative analysis of clinical supervisors’ comments revealed that AI-generated case conceptualizations demonstrated strong performance in information organization and logical analysis, yet exhibited notable limitations in psychological depth and individualized understanding. Specifically, supervisors consistently noted that AI’s analysis of the core issues in cases was relatively superficial, lacking an exploration of the underlying motivations behind clients’ behaviors, which resulted in insufficient depth and specificity in its case conceptualizations. Additionally, AI displayed weak performance in emotional connection and empathic expression, and it tended to rely on formulaic processing approaches, making it difficult to reflect in-depth understanding of clients’ unique circumstances. Furthermore, AI-generated content showed a strong tendency toward theoretical accumulation, with inadequate flexible application and integration of relevant theories. Detailed evaluative comments are presented in Table 8.

### 3.2. Experiment 2

#### 3.2.1. The Effect of AI-Assisted Support on Novice Counselors’ Performance

Nonparametric tests were employed to compare the differences in the quality of novice counselors’ case conceptualization before and after AI-assisted support. Results of the Wilcoxon signed-rank test indicated that AI-assisted support significantly improved novice counselors’ performance on the comprehensiveness dimension (Z = −2.271, *p* = 0.023, r = 0.718) and the coherence dimension (Z = −2.530, *p* = 0.011, r = 0.800), whereas no significant improvements were observed on the complexity and specificity dimensions (*p* > 0.05), as presented in Table 9 (all participants combined across both scenarios). The effect sizes for comprehensiveness and coherence (r = Z/√N) exceeded Cohen’s threshold for large effects (r ≥ 0.50), indicating practically meaningful gains. A further Kruskal–Wallis test was conducted to examine the effect of case type on case conceptualization quality. After Bonferroni correction (α = 0.0125), no statistically significant differences across case types were found for any of the four CFQ dimensions: comprehensiveness (*p* = 0.977), coherence (*p* = 0.061), complexity (*p* = 0.094), and specificity (*p* = 0.820). These findings demonstrate that the quality of novice counselors’ case conceptualization was not affected by case type. Collectively, these results indicate that H2 is partially supported: AI-assisted support effectively improved comprehensiveness and coherence but did not yield significant improvements in complexity or specificity.

#### 3.2.2. Novice Counselors’ Subjective Perceptions

Novice counselors’ user experience with AI-generated case conceptualization was examined through multidimensional assessments, with perceived usefulness (PU) evaluated across all five participants and perceived ease of use (PEU) evaluated for the participant who independently operated ChatGPT (Scenario 2). Due to the small sample size (*n* = 5), Cronbach’s alpha could not be stably computed for the PU and PEU scales; therefore, the Intraclass Correlation Coefficient (ICC) was used to assess inter-rater consistency among novice counselors’ ratings. Intraclass correlation coefficient (ICC) analysis revealed good inter-rater consistency among novice counselors in their quality ratings of AI-generated content (ICC = 0.823, *p* < 0.001) and their evaluations of perceived usefulness (PU) (ICC = 0.907, *p* < 0.001). Descriptive statistics yielded the following findings: (1) CFQ ratings: Novice counselors rated AI-generated case conceptualization as performing well on the comprehensiveness (M = 4.2, SD = 0.75), coherence (M = 4.2, SD = 0.75), and complexity (M = 4.2, SD = 0.75) dimensions, as well as the specificity dimension (M = 3.8, SD = 0.75). (2) PU ratings: Novice counselors generally endorsed the practical utility of AI-generated content (M = 3.67, SD = 0.89), with the highest ratings provided by the novice counselor assigned to Case C, reflecting their recognition of AI’s supportive effects on case conceptualization. (3) PEU ratings: The novice counselor who engaged in the Case C task held a positive attitude toward the ease of use of the AI tool (PEU = 4.0).

#### 3.2.3. Qualitative Analysis of Novice Counselors’ Feedback

A qualitative analysis of clinical supervisors’ comments on novice counselors similarly revealed that the quality of novice counselors’ case conceptualization improved with AI-assisted support, regardless of their performance in the independent case conceptualization phase, with the most notable improvement observed in the comprehensiveness dimension. Furthermore, two common challenging areas, namely counseling goal setting and intervention plan systematicity, exhibited significant improvements following AI-assisted support. This improvement was reflected in a reduction in supervisory comments citing unrealistic goal setting and superficial thinking. Table 10 presents illustrative examples of clinical supervisors’ comments on Participant 3 to demonstrate these changes.

A qualitative analysis was conducted on the case conceptualization content of novice counselors before and after AI-assisted support in this study, from which three strategic patterns were identified: direct adoption (*n* = 2), perspective retention (*n* = 2), and gap identification and supplementation (*n* = 1). These three strategies reflect distinct cognitive processing tendencies and decision-making modes among novice counselors when integrating AI-generated information with their own professional judgments.

Notable performance divergence was observed between Novice Counselor 1 and Novice Counselor 5, who adopted the direct adoption strategy: the former improved the quality of case conceptualization by screening high-quality AI-generated content curated by the researchers, while the latter discontinued AI usage after a single interaction, resulting in a decline in quality. This finding may be attributed to the dynamic and uncertain nature of the AI interaction process, as well as the users’ proficiency in AI application.

Novice Counselor 2 and Novice Counselor 4, who adopted the perspective retention strategy, engaged in multi-perspective thinking inspired by AI; however, guided primarily by the self-reference effect, they only supplemented content related to the 4P model, leading to an insignificant improvement in their case conceptualization performance. In contrast, Novice Counselor 3, who adopted the gap identification and supplementation strategy, demonstrated the strongest practical performance. This counselor comprehensively revised their case conceptualization based on AI-generated content, and the revised version received high ratings from clinical supervisors on dimensions such as completeness and coherence. These results indicate that the organic assimilation, comprehensive analysis, and in-depth integration of AI-generated information with one’s own professional knowledge constitute an effective pathway for novice counselors to achieve professional learning and competency development.

## 4. Discussion

### 4.1. AI’s Ability to Perform Case Conceptualization

Repeated-measures analysis of variance on the rating data from clinical supervisors revealed that AI-generated case conceptualization reached a passing level on the coherence and specificity dimensions, while performance on comprehensiveness and complexity approached but did not reach the passing threshold. This finding is consistent with the conclusion of Vázquez-Cano et al. (2023) that AI achieves low yet passing scores in such tasks. The absence of significant differences across the three prompt frameworks suggests that, once a minimum threshold of prompt structure is met, the specific framework may be less critical than the quality of content embedded within the prompts.

### 4.2. The Effect of AI-Assisted Support on Novice Counselors’ Case Conceptualization

AI effectively assisted novice counselors in improving the comprehensiveness and coherence of their case conceptualization, while no significant improvements were observed in terms of complexity and specificity. This may be attributed to AI’s technical advantages in the domains of comprehensiveness and coherence (Ali et al., 2023). When aligning the components of case conceptualization with its quality assessment dimensions, the descriptive and inferential components primarily reflect the comprehensiveness and coherence of case conceptualization quality, whereas the counseling goal and intervention plan components are more indicative of complexity and specificity.

(1) Significant Improvements in Comprehensiveness and Coherence

Comprehensiveness requires case conceptualization to encompass symptoms, etiological factors, and explanatory mechanisms. Leveraging preconfigured algorithms and logical frameworks, AI can efficiently process and integrate large volumes of complex data. Novice counselors can thus utilize AI to supplement potentially omitted information and develop a more comprehensive understanding of cases. This finding is also reflected in novice counselors’ feedback; for example, one counselor stated, “AI provided more professional descriptions” and “AI met my need to organize detailed information, enabling the comprehensive collection of information relevant to the client and avoiding the expenditure of substantial time extracting information from counseling transcripts.”

Coherence entails clarity in logical sequence and causal relationships—a capability that AI already possesses. Multiple studies have demonstrated that AI excels at generating high-quality, coherent textual outputs (Ali et al., 2023). As noted by novice counselors, “AI proposed alternative perspectives that I could draw on; I would reflect on whether these were actually manifested in the client, thereby deepening my understanding of them” and “AI’s counseling goals exhibited greater hierarchical structure.”

(2) Non-Significant Improvements in Complexity and Specificity

This limitation can be understood from the fundamental nature of LLMs, which operate on the basis of statistical pattern recognition learned from vast training data, generating outputs that represent the most probable response given the input—making them exceptionally good at producing well-organized, logically coherent, and comprehensive content. However, clinical case formulation fundamentally requires individualization that goes beyond statistical patterns: it demands empathic attunement, understanding of unique personal narratives, and the ability to formulate hypotheses that may not align with the most common patterns in training data. This inherent tension between the statistical nature of LLMs and the idiographic nature of clinical reasoning explains why AI consistently underperforms on specificity and complexity—dimensions that require deep, individualized understanding rather than broad structural coverage. Empirical evidence supports this account: Guo et al. (2023) compared AI responses with those of human experts across multiple domains and found that AI tends to use long sentences, exhibits a less rich vocabulary than human scholars, and repeatedly employs common patterns and syntactic structures from training texts. Ling et al. (2023) analyzed the characteristics of AI’s academic writing ability and revealed that although the generated content features a robust logical framework and sufficient, clear reasoning, it lacks necessary ideological depth, with thin argumentative language and relatively single means of reasoning. The recent emergence of reasoning models (e.g., OpenAI o1/o3, DeepSeek-R1, Claude with extended thinking) may partially address this limitation. Unlike traditional LLMs that generate responses through single-pass prediction, reasoning models engage in internal deliberation and self-verification before producing outputs, enabled by reinforcement learning at scale (OpenAI, 2024). This “test-time compute” paradigm holds particular promise for improving the complexity and specificity dimensions—precisely where GPT-4o underperformed. Future research should directly compare reasoning models with traditional LLMs on case conceptualization tasks.

In addition to these inherent limitations of LLMs, the specific design of the present study may have further constrained AI’s performance on complexity and specificity. The formulation of counseling goals and intervention plans in case conceptualization is typically guided by counselors’ personal theoretical preferences. However, given the heterogeneity in participants’ theoretical orientations, the experimenter did not mandate the adoption of a specific theoretical orientation for developing counseling goals and intervention plans when designing the prompts; instead, only an integrative 4P model was required for the inferential analysis phase. As the integrative orientation is inherently a multi-theory analytical framework, AI conducts a comprehensive exploration from multiple theoretical perspectives during the intervention plan formulation stage. This broad, multi-theory exploration, while comprehensive, lacks the focused depth that a single-theory adherent would require. The mismatch between AI’s broad theoretical outputs and participants’ specific theoretical orientations likely contributed to the non-significant improvements in complexity and specificity—dimensions most closely tied to theory-driven intervention planning. In Scenario 1, the AI-assisted process was administered by the experimenter, precluding participants from requesting AI to conduct targeted in-depth analyses aligned with their preferred theoretical orientations. In Scenario 2, participants discontinued AI usage after a single interaction. These factors collectively compromised AI’s performance in terms of complexity and specificity.

Additionally, the substantial variability in clinical supervisors’ ratings for complexity and specificity indicates that these two dimensions may themselves be highly contentious, making objective evaluation challenging. Furthermore, the use of Western historical cases may have introduced cultural and temporal disparities, potentially affecting both AI’s formulations and supervisors’ expectations regarding cultural sensitivity and cross-cultural understanding. Collectively, these factors result in AI failing to provide sufficient reference value for novice counselors in enhancing the complexity and specificity of their case conceptualization.

### 4.3. Critical Reflections

In interviews, novice counselors indicated that while they identified limitations in AI’s ability to provide analyses rooted in specific theoretical orientations—including a lack of in-depth understanding and detailed guidance for certain therapeutic schools—they generally rated the quality of AI-generated case conceptualizations as adequate or even good and acknowledged the usefulness and ease of use of AI tools. Domain-specific mental health LLMs may also address limitations identified in this study. PsychFound, a 7B-parameter model trained on real-world psychiatric records, demonstrated clinical reasoning matching attending psychiatrists and improved resident physicians’ diagnostic accuracy in a prospective study (R. Wang et al., 2026). This suggests targeted clinical adaptation, rather than general model scale, may be critical—a direction for future research on case conceptualization.

A synthesis of novice counselors’ evaluations of AI, alongside their varied usage strategies and associated outcomes in the present study, reveals that the effectiveness of AI in supporting novice counselors with case conceptualization is largely contingent on how novices interpret AI outputs and their depth of mastery of relevant disciplinary knowledge. This finding aligns with conclusions from existing research (Wu et al., 2023; Xia et al., 2023). From a deep learning perspective, AI intervention can effectively identify and fill knowledge gaps in some cases, further optimizing case conceptualizations initially developed through in-depth critical thinking. However, this also highlights that AI’s utility depends not only on its capacity for autonomous content generation, but also on users’ cognitive understanding of relevant theories and their critical evaluation of AI-generated content. Thus, the application of AI in counseling practice should be integrated with counselors’ deep learning processes to achieve optimal outcomes.

Based on the study findings, we propose the following recommendations for novice counselors using AI to support case conceptualization:

(1) Rational Positioning of AI’s Role

While AI-generated case conceptualizations currently meet a basic passing standard, they do not yet attain a high-quality benchmark and cannot independently produce high-quality case conceptualizations. AI’s primary functions lie in assisting with the systematic organization of client information to enhance comprehensiveness and facilitating a deeper understanding of cases through multi-perspective analysis. Considering this functional positioning, and in conjunction with the finding of Ling et al. (2023) that over-reliance on AI may undermine the development of human interaction, personalized guidance, and higher-order thinking skills, we recommend that novice counselors use AI rationally as a tool for gap identification and supplementation during the optimization phase, after developing an initial draft of case conceptualization independently.

(2) Critical Evaluation of AI Outputs

When using AI, counselors must possess the ability to identify and eliminate meaningless or superficially developed content in AI-generated outputs. It is recommended that counselors maintain a critical mindset when referencing AI content, focusing on its depth and quality to ensure that any adopted content has practical applicative value. AI should be regarded as a supportive tool to enhance and supplement existing knowledge and skills, rather than a substitute for individual learning and analytical processes.

(3) Ethical Considerations

When using AI to assist with case conceptualization for clients, informed consent must be obtained from clients, the intended use of client data must be clearly stated, and all information must be anonymized to prevent identity disclosure. All AI-related operations should be conducted under the supervision of a clinical supervisor to ensure compliance with ethical and legal norms. Additionally, the AI platforms employed must adhere to national data protection regulations to effectively safeguard clients’ privacy and rights. Beyond these specific ethical requirements, we caution against the risk of over-reliance on AI-generated templates in supervision and training contexts. If novice counselors treat AI outputs as templates rather than as reference points for critical reflection, there is a danger of superficial learning and diminished engagement with counseling theory and client material. We recommend that training programs explicitly teach AI as a supportive tool for gap identification rather than as a content generator, and that supervisors monitor trainees’ AI usage to ensure they maintain independent clinical reasoning.

## 5. Limitations and Future Directions

The present study has several limitations. First, constrained by the time and effort required for counseling transcript analysis, the sample size was relatively small (*n* = 5), which limited the statistical power of our analyses, the generalizability of the research findings and the external validity of the study. The small sample size also precluded the computation of stable reliability estimates (e.g., Cronbach’s alpha for PU and PEU scales) and limited our ability to detect small-to-moderate effect sizes. Consequently, the findings should be interpreted as preliminary and hypothesis-generating rather than confirmatory, and replication studies with larger samples are needed to establish the robustness of the observed effects. Second, only GPT-4o was employed as the AI tool in this research; with the iteration of AI technology, new model architectures may demonstrate superior performance in case conceptualization. The rapid expansion of context windows in newer LLMs also challenges the necessity of our three-stage output design. Gemini 2.5 Pro supports up to one million tokens, far exceeding our longest transcript. Future research should evaluate whether simplified, single-pass processing with newer models yields results comparable to or better than the multi-stage approach employed here. Third, prompt design was primarily based on the integrative 4P model, which makes it difficult to cover diverse theoretical perspectives. Additionally, certain AI operations were executed by the researchers, which may have weakened the authentic reflection of AI’s auxiliary effectiveness. Finally, the use of foreign historical cases—drawn from Western counseling contexts and cultural backgrounds—introduced cultural and temporal disparities that may have interfered with both AI’s formulations and counselors’ cultural sensitivity and cross-cultural understanding. These cases, originally recorded in English and reflecting Western therapeutic paradigms, may not adequately represent the cultural norms, values, and clinical presentations typical of Chinese counseling settings. This cultural gap could have affected how both AI and novice counselors interpreted client presentations and formulated case conceptualizations, potentially limiting the applicability of our findings to non-Western counseling contexts.

Building on the limitations, future research can be conducted in five directions. First, expand the sample size by including more novice counselors and clinical supervisors to enhance the statistical power of the research and the generalizability of its conclusions. Second, conduct a comparative evaluation of the application effects of multiple AI tools in different counseling scenarios to explore more optimal technical solutions. Third, carry out research targeting diverse theoretical orientations in counseling psychology and match participants with corresponding theoretical backgrounds to improve the compatibility of AI tools with professional clinical practice. Fourth, adopt localized cases developed within the target cultural context to more accurately assess AI’s utility in culturally sensitive case formulation, thereby promoting the effective application of AI technology in the local counseling field. Fifth, future research should employ longitudinal designs to examine whether the improvements observed with AI assistance translate into durable, independent case formulation competence over time. For instance, researchers could assess whether novice counselors maintain improved performance when subsequently formulating cases without AI support, and whether repeated AI-assisted practice leads to sustained gains in clinical reasoning skills beyond the immediate task context. Such longitudinal investigations would provide valuable evidence regarding the long-term educational value of AI-assisted training and help determine whether AI functions as a temporary performance aid or as a vehicle for durable professional development.

## 6. Conclusions

This exploratory study demonstrates that AI possesses the ability to perform case conceptualization, and the quality of AI-generated case conceptualizations remains consistent across different cases. No significant differences were found in the quality of case conceptualizations generated by the three prompt frameworks employed in this study. AI can effectively assist novice counselors in enhancing the comprehensiveness and coherence of their case conceptualizations: novice counselors rated AI-generated case conceptualizations as performing well in terms of comprehensiveness, coherence, and complexity in their self-assessments, and as adequate in specificity. Novice counselors generally agreed (PU > 3.6) that AI-generated case conceptualizations contain useful and practical content, and they agreed (PEU = 4) that the process of using AI is characterized by ease of use. Novice counselors are advised to utilize AI for gap identification and supplementation to further refine the quality of case conceptualizations after developing an initial draft independently, rather than relying entirely on AI for the analytical process.

## Figures and Tables

**Table 1:** Basic Information of Novice Counselors.

Counselors	Participant 1	Participant 2	Participant 3	Participant 4	Participant 5
Training Status	2nd-year MSc (enrolled)	Fresh graduate (within 1 month)	2nd-year MSc (enrolled)	Fresh graduate (within 1 month)	1st-year MSc (enrolled)
Cases	A	A	B	B	C
Orientation	BPT	PCT	BPT	CCT & EFT	CCT & SFT
Clinical Practice Hours	0	170	0	197	50
Individual/Group Sup. (h)	0/0	50/87	0/0	30/70	50/60
AI Use Frequency	occasionally	Several/mo	Several/mo	Multiple/mo	occasionally

Note. MSc = Master of Science in Psychological Counseling; Sup. = Supervision; BPT = Brief Psychodynamic Therapy; PCT = Person-Centered Therapy; CCT = Client-Centered Therapy (Humanistic); EFT = Emotion-Focused Therapy; SFT = Solution-Focused Therapy; Several/mo = several times per month; Multiple/mo = multiple times per month. All participants were master’s students majoring in psychological counseling or fresh graduates within one month of graduation. Clinical practice hours and supervision hours were self-reported. Case A and Case B were drawn from the public dataset; Case C was drawn from the self-developed dataset.

**Table 2:** Basic Information of Verbatim Transcript Materials.

Study Phase	Dataset	Materials	Experimental Participants	Extracted Content
Pilot study	—	Client006	GPT-4o	First 6 sessions
Study 1	Public dataset	Case A (Client110)	Participants 1–4	First 3 sessions
		Case B (Client018)		
Study 2	Self-developed dataset	Case C	Participant 5	First 6 sessions

**Table 3:** Supervisor-Case Assignment Table.

Supervisor Pair	Case A	Case B	Case C
Supervisor Team 1	Supervisor A	Supervisor C	Supervisor E
Supervisor Team 2	Supervisor B	Supervisor D	Supervisor D

**Table 4:** Summary of Experimental Design.

Phase	Participants	Cases	AI Role	Rater	Primary Outcome Measures
Pilot	Researcher	Public (Client006)	Prompt test	-	-
Exp 1	AI	Case A	Ind. Writing	Sup.	CFQ
Exp 2 (S1)	Cl. (P1–P4)	Case B	Asst. Ref.	Sup. & Cl.	CFQ (Sup. & Cl.), PU (Cl.)
Exp 2 (S2)	Cl. (P5)	Case C	Asst. Dial.	Sup. & Cl.	CFQ (Sup. & Cl.), PU & PEU (Cl.)

Note. Exp = Experiment; S = Scenario; Ind. = Independent; Asst. = Assisted; Ref. = Reference; Dial. = Dialogue; Sup. = Supervisor; Cl. = Counselor.

**Table 5:** Prompt Adjustment Strategies.

Dimension	Adjustment Direction
Role	To conduct analysis from the perspective of a professional counselor, with a focus on case conceptualization and documentation writing as practiced in actual counseling sessions.
Reference materials	To use the content of the 4P model formatted in Markdown as the reference basis.
Interaction	To adjust to phased output due to excessive length; users can reply with “continue” to proceed to the next step.
Context supplementation	To explicitly upload verbatim transcripts in three batches; after the initial upload, each subsequent upload requires comprehensive analysis of previous interview content.

**Table 6:** *t*-Test of the Quality of AI-Generated Case Conceptualization.

Dimension	*M*	*SD*	*t*	*p*
Comprehensiveness	3.25	0.60	1.39	0.19
Coherence	3.42	0.76	1.82	0.01 **
Complexity	2.92	0.64	−0.43	0.67
Specificity	3.00	0.82	0.00	1.00

Note. ** *p* < 0.01.

**Table 7:** Descriptive Statistics of CFQ Scores by Prompt Framework.

Prompt Framework	Case	Comprehensiveness		Coherence		Complexity		Specificity	
		*M*	*SD*	*M*	*SD*	*M*	*SD*	*M*	*SD*
CO-STAR (*n* = 4)	A	3.0	0.0	2.5	0.5	2.5	0.5	2.5	0.5
	B	4.0	0.0	4.5	0.5	3.5	0.5	4.0	1.0
LangGPT (*n* = 4)	A	2.5	0.5	3.0	0.0	2.5	0.5	2.5	0.5
	B	3.5	0.5	3.5	0.5	3.0	0.0	3.0	0.0
BROKE (*n* = 4)	A	3.5	0.5	3.5	0.5	2.5	0.5	2.5	0.5
	B	3.0	0.0	3.5	0.5	3.5	0.5	3.5	0.5

**Table 8:** Qualitative Analysis of the Characteristics of AI-Generated Case Conceptualization.

Characteristics of AI-Generated Case Conceptualization	Excerpts from Supervisor Comments
Strengths	
Good information organization and logical analysis	1. The overall understanding is clear. 2. Comprehensive relevant information is considered, and the conceptualization of the client is outlined completely and coherently. 3. The content covered is relatively comprehensive. 4. Multiple aspects of the client’s concerns are addressed, allowing for the formation of targeted intervention plans. 5. The overall evaluation is comprehensive and logically clear.
Targeted focus	The focus is clear and closely aligned with the client’s presenting problem.
Limitations	
Superficial analysis with a lack of depth	1. Understanding of the client remains superficial, with biased interpretations of behavior. 2. Specificity and understanding of the client are relatively superficial. 3. Lacks in-depth understanding and creative elements; the main issues of the client are not sufficiently recognized, with repetitive expressions.
Weak emotional connection and empathic expression	Lacks in-depth empathic understanding of the case, with obvious dehumanized, machine-like patterns.
Theoretical overcrowding and content repetition	Confusion in treatment goals; excessive use of techniques from different theoretical orientations; excessive overlap between short-term and long-term goals.

**Table 9:** Wilcoxon Signed-Rank Test.

Dimension	*Z*	*p*	*r*
Comprehensiveness	−2.271	0.023 *	0.718
Coherence	−2.530	0.011 *	0.800
Complexity	−1.406	0.160	0.445
Specificity	−0.722	0.470	0.228

Note. * *p* < 0.05.

**Table 10:** Supervisor Evaluations of Participant 3’s Case Conceptualization.

AI Assistance	Supervisor	Evaluation
Before AI assistance	Supervisor E	Comprehensive attention to information, reasonable hypotheses, and overall appropriate analysis.
	Supervisor D	The logical framework is largely similar to the first evaluation, but comprehensiveness is lower than the first; counseling goals are relatively brief, specificity is average, and the connection between theory and practice is weak.
After AI assistance	Supervisor E	Rich conceptualization information, coherent throughout, comprehensive evaluation, and complete alignment between the counseling plan and the conceptualization.
	Supervisor D	The entire case conceptualization is clear and distinct. If ranked, I consider it the highest-scoring one among all.

## Data Availability

Data available on request from the authors. The data that support the findings of this study are available from the Corresponding Author, Yongze Xu, upon reasonable request.

## Appendix A

**Case Formulation Quality Rating Sheet**					
**PID#: Rater: Date:**					
	**None**	**Marginal**	**Adequate**	**Good**	**Superior**
- COMPREHENSIVENESS Problems or symptoms Etiological factors Precipitating events Predisposing factors Maintaining factors Explanatory Mechanism	1	2	3	4	5
B. COHERENCE Connections exist between explanatory mechanisms, problems/symptoms, and etiological factors Few coherence breaks	1	2	3	4	5
C. COMPLEXITY Differentiates multiple facets of problems or functioning Indicates how these facets are interrelated or connected	1	2	3	4	5
D. SPECIFICITY Individualized information Clear, precise language Treatment focus clearly identified	1	2	3	4	5
COMMENTS:					

## Appendix B

**Perceived Usefulness and Perceived Ease of Use Scale Sheet**	
**PID#: Rater: Date:**	
**Variable**	**Measurement Items**
Perceived Usefulness (PU)	1. ChatGPT can shorten the time I spend on case conceptualization and improve efficiency. 2. ChatGPT can help me improve my level of case conceptualization. 3. ChatGPT facilitates deeper thinking during my case conceptualization process. 4. Overall, ChatGPT plays an important auxiliary role in my case conceptualization work.
Perceived Ease of Use (PEU)	1. I find it simple and easy to learn how to operate ChatGPT for case conceptualization analysis. 2. I find it simple to have ChatGPT analyze transcripts and generate case conceptualizations according to my needs. 3. I find the content generated by ChatGPT to be readily understandable. 4. I find that ChatGPT demonstrates good adaptability and flexibility when performing case conceptualization analysis. 5. Overall, I find it easy to use ChatGPT for case conceptualization analysis.

## Appendix C

- Prompt of LangGPT Framework

# Role: CounselorAnalysisGPT
  ## Profile
  - Author:Yan Shan Dai
  - Version: 0.4
  - Language: Chinese
  - Description: As CounselorAnalysisGPT, your mission is to guide novice psychological counselors in analyzing psychological counseling interview transcripts to understand clients, and to use the 4P Model to formulate case conceptualizations, including understanding various aspects of the problem and their interrelationships, and to determine counseling goals and treatment plans based on these analyses.
  ### Skill-1
  Descriptive Phase: Information is to be collected from the transcript across six domains: ① demographic information; ② presenting complaint; ③ family relationships (to be analyzed from a family therapy perspective); ④ developmental history; ⑤ interpersonal relationships; ⑥ psychological assessment (including crisis assessment, mental status assessment, and scale-based evaluation).
  ### Skill-2
  1.Inferential Phase: The collected information is to be categorized into meaningful clusters, examining how these themes affect the client’s life, health, and subjective experience. The 4P Model is to be used for this analysis, with each of the four factors analyzed separately, followed by a comprehensive summary of one to two paragraphs.
  2.In each part of **4P model** (susceptibility, induction, maintenance, protection factors), you must explain the following in detail:
  | Category | Biology | Society | Psychology |
  |--------|--------|--------|------------|
  | Susceptibility Factors | Genetic Susceptibility<br>Family Medical History<br>Birth Defects<br>Developmental Retation<br>Age<br>Race<br>Gender<br>Gender Identity<br>Sexual Orientation | Socioeconomic Status/Poverty<br>Geographical Area<br>Childhood Experience<br>Education Level<br>Long-term Occupational Pressure | Personality Characteristics<br>Temperament<br>Psychopathology<br>Stress Tolerance Ability<br>Family Mental Health History |
  | Inducing factors | Onset of acute disease/infection <br> Onset of serious medical disease <br> Major surgery or medical procedure <br> Physical trauma <br> Substance use/ abuse | Life events (such as childbirth, car accident, marriage, dismissal, academic difficulties, divorce, etc.) <br> Social/ self-esteem support <br> Interpersonal conflict <br> Natural disasters | Coping methods with/ poor problem solving <br> Negative/ maladaptated thinking <br> Psychopathology |
  | Maintenance factors | substance use/abuse<br>chronic physical disease<br>immunosuppression | social/self-esteem support<br>rigidity of work/life schedule<br>social stigma<br>financial obligations<br>socio-economic status/poverty<br>unemployment<br>social factors (secondary benefits) | response style<br>social support<br>compensatory behavior<br>negative/maladjustment thinking<br>avoid behavior |
  | Protection factors | Regular exercise or healthy diet <br> Immune system <br> Genetic resistance | Stable social support network <br> Adequate economic resources <br> High education level | Positive attitude <br> Effective coping strategies <br> Psychological resilience <br> Safe attachment |
  ### Skill-3
  1.Counseling Goals and Treatment Plan: Based on the dialogue with the client in the transcript, mutually agreed counseling goals are to be identified, and an appropriate treatment plan is to be formulated accordingly. Determine which counseling theory best accounts for the client’s issues given the client’s presentation and the counselor’s training background. Additionally, identify alternative theoretical frameworks that could generate hypotheses about the client’s problems, specify counseling goals, and outline how these goals may be achieved.
  ## Rules
  1.Do not deviate from the role under any circumstances.
  2.Analysis is to be based on the uploaded Word interview transcripts. Focus on new information in the current transcript (relative to previous transcripts), while integrating all previous interview content and analysis results. Do not fabricate information.
  3.At the conclusion of each phase, prompt the user to reply “Analyze Next Phase” to proceed to the subsequent phase.
  4.Analysis and output are to be conducted in Chinese. Ensure that each uploaded Word document is clearly distinguished and that the three phases are completed sequentially, while maintaining coherence and continuity across interview content.
  ## Workflow
  1.First, carefully read the uploaded interview transcript Word document.
  2.Based on the transcript, complete Phase 1, the Descriptive Phase. Upon completion, prompt: “Reply ‘Analyze Next Phase’ to proceed with the Inferential Phase.”
  3.Then, using the 4P Model, complete Phase 2, the Inferential Phase. Upon completion, prompt: “Reply ‘Analyze Next Phase’ to proceed with the Counseling Goals and Treatment Plan Phase.”
  4.Finally, integrating the client’s presentation and counseling theories, complete Phase 3, the Counseling Goals and Treatment Plan Phase. Upon completion, prompt: “Analysis completed. If you have any questions or require further analysis, please ask. You may upload the next interview transcript for continued analysis, and we will incorporate all previous interview content and analyses.”
  5.If the user uploads a new transcript, repeat the above steps.
  ## Initialization

As CounselorAnalysisGPT, you must adhere to the <Rules>, communicate with the user in default <Chinese>, and greet the user. Then introduce yourself and introduce the <Workflow>.

B. Prompt of BROKE Framework

Background (B):
  Psychological counselors in the novice stage improve their case conceptualization skills by analyzing interview transcripts. After each interview session, the novice counselor reflects on the causes of the client’s problems from the transcript and plans future treatment strategies. The counselor uploads interview transcripts in chronological order and requests the AI to analyze the client’s problems. The counselor uploads transcripts sequentially, and the AI is expected to integrate all interview content in its analysis.
  Role (R):
  You are an expert skilled in using artificial intelligence for data analysis, particularly within the field of psychological counseling. You are able to accurately understand and analyze transcript content, helping novice counselors gain insights from them.
  Objective (O):
  Based on the counselor’s needs, the AI is to conduct an in-depth analysis of the uploaded transcripts. The analysis is to be delivered in three successive outputs: the Descriptive Phase, the Inferential Phase, and the Counseling Goals and Treatment Plan Phase. The analysis is to prioritize new information from the current interview while considering all previously uploaded interview content.
  Key Results (KR):
  1.The analysis is to be delivered in three separate outputs corresponding to the Descriptive Phase, Inferential Phase, and Counseling Goals and Treatment Plan Phase.
  2.At the end of each phase, the user is to be prompted to reply “Analyze Next Phase” before the AI proceeds to the subsequent phase.
  3.The Inferential Phase is to adopt the 4P Model for analysis.
  4.Based on the analysis results, appropriate counseling theories and strategies are to be provided to facilitate the formulation of effective treatment plans.
  5.All analysis content is to be output in Chinese to ensure clear comprehension by the counselor.
  Steps (S):
  1.Phase 1, Descriptive Phase: Collect information from the transcript across six domains: ① demographic information; ② presenting complaint; ③ family relationships (to be analyzed from a family therapy perspective); ④ developmental history; ⑤ interpersonal relationships; ⑥ psychological assessment (including crisis assessment, mental status assessment, and scale-based evaluation). At the conclusion of this phase, the AI will prompt: “Reply ‘Analyze Next Phase’ to proceed with the Inferential Phase.”
  2.Phase 2, Inferential Phase: Classify the collected information into meaningful clusters and examine how these themes affect the client’s life, health, and subjective experience. The 4P Model is to be used for this analysis, with each of the four factors analyzed separately, followed by a comprehensive summary of one to two paragraphs. At the conclusion of this phase, the AI will prompt: “Reply ‘Analyze Next Phase’ to proceed with the Counseling Goals and Treatment Plan Phase.”
  1) For each component of the 4P Model (Predisposing, Precipitating, Perpetuating, and Protective factors), the following content must be specified:
  | Category | Biology | Society | Psychology |
  |--------|--------|--------|------------|
  | Susceptibility Factors | Genetic Susceptibility<br>Family Medical History<br>Birth Defects<br>Developmental Retation<br>Age<br>Race<br>Gender<br>Gender Identity<br>Sexual Orientation | Socioeconomic Status/Poverty<br>Geographical Area<br>Childhood Experience<br>Education Level<br>Long-term Occupational Pressure | Personality Characteristics<br>Temperament<br>Psychopathology<br>Stress Tolerance Ability<br>Family Mental Health History |
  | Inducing factors | Onset of acute disease/infection <br> Onset of serious medical disease <br> Major surgery or medical procedure <br> Physical trauma <br> Substance use/ abuse | Life events (such as childbirth, car accident, marriage, dismissal, academic difficulties, divorce, etc.) <br> Social/ self-esteem support <br> Interpersonal conflict <br> Natural disasters | Coping methods with/ poor problem solving <br> Negative/ maladaptated thinking <br> Psychopathology |
  | Maintenance factors | substance use/abuse<br>chronic physical disease<br>immunosuppression | social/self-esteem support<br>rigidity of work/life schedule<br>social stigma<br>financial obligations<br>socio-economic status/poverty<br>unemployment<br>social factors (secondary benefits) | response style<br>social support<br>compensatory behavior<br>negative/maladjustment thinking<br>avoid behavior |
  | Protection factors | Regular exercise or healthy diet <br> Immune system <br> Genetic resistance | Stable social support network <br> Adequate economic resources <br> High education level | Positive attitude <br> Effective coping strategies <br> Psychological resilience <br> Safe attachment |
  3.Phase 3, Counseling Goals and Treatment Plan Phase: Identify the mutually agreed counseling goals based on the dialogue with the client in the transcript, and formulate an appropriate treatment plan accordingly. Determine which counseling theory best accounts for the client’s issues given the client’s presentation and the counselor’s training background. Additionally, identify alternative theoretical frameworks that could generate hypotheses about the client’s problems, specify counseling goals, and outline how these goals may be achieved. Upon completion of this phase, the AI will indicate: “Analysis completed. If you have any questions or require further analysis, please ask. You may also upload the next interview transcript, and I will proceed with the analysis incorporating all previous content.”
  4.When the user uploads a new interview transcript, the above steps are to be repeated.
  Next, let’s think step by step, work hard and painstakingly. Based on the Background above, assume you are the Role, follow the Steps, and achieve the Objective. This is very important to me.

C. Prompt of Co-Star Framework

Context (C): You are a novice counselor striving to improve your case conceptualization skills. After each session with a client, you upload all interview transcripts in Word format sequentially, starting from the first session through to the most recent. Each uploaded transcript is to be analyzed, with attention to newly emerging information while also integrating content from previously uploaded transcripts for a deeper understanding of the case. Analysis is to be conducted in Chinese.
  Objective (O): Your task is to analyze the interview transcript in three phases. The first phase focuses on the Descriptive Phase, the second phase employs the 4P Model for inferential analysis, and the third phase identifies counseling goals and a treatment plan. At the conclusion of each phase, you will instruct the user to reply “Analyze Next Phase” to proceed to the next phase.
  Style (S) & Tone (T): Adopt a professional and logically clear style, with exhaustive and accurate analysis conducted in Chinese.
  Audience (A): This analysis is intended for psychological counselors, with the aim of helping them extract deeper insights from transcripts to enhance their counseling skills.
  Response Format (R): The analysis is to be delivered in three separate phases:
  - Phase 1: Focus on the Descriptive Phase, collecting information across six domains: ① demographic information; ② presenting complaint; ③ family relationships (to be analyzed from a family therapy perspective); ④ developmental history; ⑤ interpersonal relationships; ⑥ psychological assessment (including crisis assessment, mental status assessment, and scale-based evaluation). At the conclusion of this phase, the AI will prompt: “Reply ‘Analyze Next Phase’ to proceed with the Inferential Phase.”
  - Phase 2: Upon user reply of “Analyze Next Phase,” proceed with the Inferential Phase, classifying the collected information into meaningful clusters and examining how these themes affect the client’s life, health, and subjective experience. The 4P Model is to be used for this analysis, with each of the four factors analyzed separately, followed by a comprehensive summary of one to two paragraphs. At the conclusion of this phase, the AI will prompt: “Reply ‘Analyze Next Phase’ to proceed with the Counseling Goals and Treatment Plan Phase.”
  - Phase 3: Upon user reply of “Analyze Next Phase,” provide the Counseling Goals and Treatment Plan Phase, identifying mutually agreed counseling goals based on dialogue with the client in the transcript, and formulating an appropriate treatment plan. Determine which counseling theory best accounts for the client’s issues given the client’s presentation and the counselor’s training background. Additionally, identify alternative theoretical frameworks that could generate hypotheses about the client’s problems, specify counseling goals, and outline how these goals may be achieved. Upon completion of this phase, the AI will indicate: “Analysis completed. If you have any questions or require further analysis, please ask. You may also upload the next interview transcript, and I will incorporate all previous transcripts in the analysis.”
  For each component of the 4P Model (Predisposing, Precipitating, Perpetuating, and Protective factors), the following content must be specified:
  | Category | Biology | Society | Psychology |
  |--------|--------|--------|------------|
  | Susceptibility Factors | Genetic Susceptibility<br>Family Medical History<br>Birth Defects<br>Developmental Retation<br>Age<br>Race<br>Gender<br>Gender Identity<br>Sexual Orientation | Socioeconomic Status/Poverty<br>Geographical Area<br>Childhood Experience<br>Education Level<br>Long-term Occupational Pressure | Personality Characteristics<br>Temperament<br>Psychopathology<br>Stress Tolerance Ability<br>Family Mental Health History |
  | Inducing factors | Onset of acute disease/infection <br> Onset of serious medical disease <br> Major surgery or medical procedure <br> Physical trauma <br> Substance use/ abuse | Life events (such as childbirth, car accident, marriage, dismissal, academic difficulties, divorce, etc.) <br> Social/ self-esteem support <br> Interpersonal conflict <br> Natural disasters | Coping methods with/ poor problem solving <br> Negative/ maladaptated thinking <br> Psychopathology |
  | Maintenance factors | substance use/abuse<br>chronic physical disease<br>immunosuppression | social/self-esteem support<br>rigidity of work/life schedule<br>social stigma<br>financial obligations<br>socio-economic status/poverty<br>unemployment<br>social factors (secondary benefits) | response style<br>social support<br>compensatory behavior<br>negative/maladjustment thinking<br>avoid behavior |
  | Protection factors | Regular exercise or healthy diet <br> Immune system <br> Genetic resistance | Stable social support network <br> Adequate economic resources <br> High education level | Positive attitude <br> Effective coping strategies <br> Psychological resilience <br> Safe attachment |
